# From Diagnosis to Management; Mucocele of Stump Appendicitis, Extremely Rare Finding in an Uncommon Surgical Disease: Literature Review

**DOI:** 10.1155/2021/8816643

**Published:** 2021-02-11

**Authors:** Syed Muhammad Ali, Mohannad Al-Tarakji, Fakhar Shahid, Amjad Salah Qabani, Amjad Ali Shah, Khalid Ahmed, Muhammad Burhan Khan, Inamullah

**Affiliations:** ^1^Department of Acute Care Surgery, Hamad Medical Corporation, Doha, Qatar; ^2^Department of Surgery, Hamad Medical Corporation, Doha, Qatar

## Abstract

Mucocele of the appendix is the accumulation of mucoid material in the appendiceal lumen. Although the terminology is imprecise, as it does not differentiate between the benign and malignant nature of the condition, preoperative recognition is imperative as spillage of the mucus during surgical handling can result in grave complications like pseudomyxoma peritonei. Mucocele developing in a stump of the appendix, i.e., a remnant of appendiceal tissue after surgical removal of an inflamed organ, is an extremely uncommon phenomenon, as not many cases are reported in the literature. In this review, all cases reported in English literature are discussed.

## 1. Introduction

Mucocele developing into an appendiceal stump is an uncommon lesion, as only a few cases are reported. Recognition of this condition can be challenging because of its rarity and nonspecific symptoms [1–4]. The surgical procedure should be carried out with utmost caution, whether it is diagnosed preoperatively or encountered incidentally during the surgery, as the manipulation can result in mucus release into the peritoneum leading to drastic complications [1–5]. Due to the rarity of this condition, a comprehensive review of all reported cases is described [1, 6, 7].

## 2. Methods

A thorough search of English literature was carried out with terms including stump, stump appendicitis, mucocele, and mucocele in stump in PubMed, Embase, and Google scholar and the references of all articles. More than 200 cases are reported in English literature for stump appendicitis after an initial appendectomy in the past and then developing into one of the complications like a mucocele in the stump. The data of all the articles were collected including history, examination, radiological findings, especially CT (computed tomography), operative details, and histopathology in a tabulated form.

## 3. Results

Ten cases of mucocele in the stump of the appendix were found in English literature. Tables 1 and 2 show the age, clinical features, time after index/initial appendectomy, radiological investigations with findings, operative procedure, and follow-up of all the cases. Age of the patients ranged from 27 to 79 years. Time of presentation of the mucocele in stump varied as early as four years to the latest by 40 years after the first surgery. Most of the patients presented with right lower quadrant abdominal pain or mass. All the patients were diagnosed preoperatively by imaging, and CT abdomen was the investigation of choice except one author who mentioned typical finding in ultrasound abdomen, accurately diagnosing the condition. Right hemicolectomy was the standard surgical procedure carried out in all but two patients in whom stump appendectomy with excision of mucocele was accomplished. The malignancy was ruled out partly by the size in one and frozen section in another patient in whom right hemicolectomy was not performed. One case of papillary cystadenocarcinoma was reported with peritoneal deposits, and another was mucinous cystadenocarcinoma. Follow-up was reported in 4 patients only, where the patients remained well between 1.5 and 4 years.

## 4. Discussion

Although first mentioned by Karl Freiherr von Rokitansky in 1842, Fere in 1877 coined the term mucocele and described it as appendicular dilatation secondary to accumulation of mucinous material in the lumen [8–14]. Factors, mentioned earlier by Rasch, leading to mucocele can be stenosis of appendiceal lumen with unabated mucus production and an absence of infection [8, 9, 13]. The remnant of appendix remaining attached to cecum after surgical removal of inflamed organ is called “stump”. It is usually less than 0.5 cm and does not lead to any further problem; however, in rare cases, it may develop inflammation when it is known as stump appendicitis. The first case of stump appendicitis was described by De Ruyter in 1945 (only around 200 cases have thus been reported in the English literature) [15–17].

The incidence of appendiceal mucoceles has variously been reported as 0.07–0.3% of all the appendectomy specimen [11, 12, 18] and that of appendiceal stump is further rare, as we collected and reviewed 10 cases in the literature [8].

Following mechanisms are proposed for the development of appendicular mucoceles and possibly stump mucoceles as well (World Health Organisation classification) [3, 12, 19, 20].

1. Obstruction of the proximal end secondary to inflammation and confinement of mucus. Normal or ulcerated appendiceal mucosa lines the mucocele, and these are known as retention cysts [12, 21], simple mucocele [12, 22], or ectasia of the appendix [12, 20].
2. Mucosal lining may change as seen in hyperplastic polyp of the colon [20].
3. Sometimes, lining of the mucocele is composed of atypical (papillary epithelium) reminiscent of adenomatous polyp of the large bowel [12].
4. Presence of cystadenocarcinoma where the epithelium is the same as in cystadenoma but demonstrates the features of stromal invasion by the neoplastic cells, and there may be cells in peritoneal deposits as mentioned by the Higa and others [9, 20, 23, 24].
5. Mucocele may result when an invasive adenocarcinoma (mucus secreting) affects the appendix [12, 19].

Hung mentioned obstruction of the appendiceal lumen by endometriosis and further exceptionally by inspissated mucus in cases of cystic fibrosis [25, 26]. These are the mechanisms whereby mucoceles may develop after appendectomy possibly by obstruction of the distal end of the stump, and one protective mechanism suggested is to leave a smaller stump usually <5 mm from the base [25].

Clinical manifestations are nonspecific; therefore, diagnosis is not usually straightforward. History of past appendectomy also poses difficulty and complexity in diagnosis. There is a reported delay of 4–30 years in diagnosis after the index appendectomy [7]. Patients often present with right lower quadrant abdominal pain, a slowly enlarging mass in the abdomen [12, 21, 27], unexplained and unintentional weight loss, nausea, vomiting, or bleeding per rectum. A quarter of the patients may have clinical features indistinguishable from acute appendicitis [3, 6, 27]. There are higher chances of perforations in cases of stump appendicitis, as mentioned by Liang, Thomas, and Roche-Nagle [17, 21, 28, 29]. In some cases, there is a history of slowly growing abdominal mass [12, 21, 27] that can be mistaken as duplication cysts, mesenteric cysts, abscess/hematoma, urachal cyst, or lymphangioma [1, 13, 30–32]. Occasionally, the mucoceles may be an incidental finding on exploration of the abdomen, radiological investigation, or endoscopic procedures carried out for other reasons [10, 21]. Connor mentioned if the mucoceles are symptomatic, then there is a likelihood of harboring malignancy [33]. The size has been shown to be related to malignant potential, and lesions <2 cms are mostly benign and >6 cms are usually cystadenoma or cystadenocarcinoma. [34].

There has been an association of the appendiceal mucoceles with increased incidence of colorectal neoplasms and to a lesser extent ovarian, endometrial, prostate, and other gastrointestinal malignancies, as reported by Fujiwara et al. [35]. Therefore, surveillance colonoscopy is recommended in patients diagnosed with and histopathology proven cystadenoma [3]. As mentioned, the most common presentation is vague lower abdominal pain and mass in the lower abdomen with a history of appendectomy in the previous years. One rare presentation of stump mucocele was right-sided inguinal hernia secondary to pseudomyxoma peritonei [20] and a mass extending from abdomen to thigh through the right femoral canal [1], possibly by extravasation of the mucus from appendicular mucocele into the retroperitoneum. A few patients may have a history of weight loss and altered bowel habits [6]. Certain complications may arise like intussusception, bleeding, perforation leading to peritonitis, rupture, and pseudomyxoma peritonei, which, although, are rare but can be fatal [6]. In cases of pseudomyxoma secondary to mucocele (of appendix or stump), the abdomen is filled with mucus and there might be presence or absence of epithelial cells, and according to Aho, the cellular component carries a poor prognosis [12, 21]. Yeong et al. reported a case of stump mucocele harboring papillary cystadenocarcinoma, as described by the Ozgur in their patient as well [6, 12].

Preoperative localization along with characterization is important in decision-making and alarms the operating surgeon to be extremely careful in not spilling the contents in the peritoneal cavity. Spontaneous rupture has been reported leading to pseudomyxoma and signifies high chances of malignancy [4, 6]. Notable diagnostic modalities include ultrasound, barium enema, colonoscopy, and CT abdomen [2, 3, 6, 11, 12]. Typical ultrasound findings include multiple echogenic layers in an anechoic or varied echotextures cystic mass known as “onion-skin sign” as described by Caspi et al. [36]. CT abdomen, especially the multidetector (MDCT), is the radiological investigation of choice for diagnosis and further evaluation. A mass in the right lower quadrant of abdomen with mixed CT attenuation (due to mucin contents) located in close proximity and showing continuity to cecum along with calcification of the wall provides sufficient evidence to diagnose stump mucocele [2, 3, 37, 38]. Mural calcification is seen in half of the lesions; therefore, the distinction between mucocele and cecum is challenging in larger masses, but coronal sections are especially helpful in delineating the origin [4, 33]. Barium enema was used frequently in the past for diagnosing colonic pathologies, and in one earliest case, it showed smooth deformity or indentation of the inferomedial cecal wall, implying a pressure effect on the terminal ileum [8]. Colonoscopy can likewise demonstrate a bulge produced by the external compression of the cecum [11].

In the recent era, MDT (multidisciplinary teams) meetings are suggested to improve the management and outcome of all, particularly neoplastic diseases, so every case diagnosed preoperatively should be discussed, and appropriate management strategy is individualized. A surgical procedure in the form of limited ileocecal resection or right hemicolectomy is suggested as an optimum treatment for the mucocele of the stump appendix [1, 3, 6, 8, 10, 11, 25]. Open surgical exploration was performed in almost all cases, but laparoscopic resection has been suggested by Cama in which a 3∗2 cms stump mucocele was resected successfully. Almost all authors have suggested and stressed on the extreme care for not damaging the specimen during handling as the sequelae can be disastrous. Some have argued as the right hemicolectomy does not offer a survival advantage, so appendectomy or cecectomy with negative margins is a preferable method of treatment [34]. El Ajmi et al. preferred to perform right hemicolectomy and lymph node dissection, as there was suspicion of malignancy [10]. Histopathology of all the cases reviewed was benign except for two. Since there are only a few cases reported, a guideline could not be formulated though right hemicolectomy is a plausible choice in all but very small, localized lesions. The follow-up for some of the benign cases have been reported, but none has been mentioned for the two malignant patients who were operated by radical resections, and it is recommended in all patients as they may develop any other type of gastrointestinal malignancies.

## 5. Conclusion

Mucoceles in appendix are rarely encountered and in stump of appendix is further infrequent. A few cases are reported, and the most common presentation is with vague lower abdominal pain or slowly growing mass. US and CT scans are diagnostic in all cases, and surgical removal in the form of right hemicolectomy is the treatment of choice although stump appendectomy can be considered in smaller lesions. Resection should be carried out with utmost precaution to remove the mass intact as rupture may lead to catastrophic consequences. Multidisciplinary teams can play a vital role in managing the individual patients, and postoperative surveillance is needed as there are chances of other gastrointestinal neoplastic lesions in the future.

## Figures and Tables

**Table 1:** Review of literature of appendiceal mucocele in stump appendix.

No.	Author	Age	Gender	Presentation	Interval between appendectomy	Open vs. lap	Time of diagnosis
1	Rasch and Strange [8]	54	Male	Right lower abdominal pain	4 years	Open	Post-op

2	Ozgür et al. [6]	75		Abdominal pain, nausea and vomiting, and mass on examination	10 years	Not mentioned	Pre-op

3	Cama Jitoko K [21]	27	Female	Chronic abdominal pain in RIF for 12 months	18 years	Open	Post-op

4	El Ajmi et al. [10]	54	Female	3-week history of pain in the right lower quadrant of the abdomen	20 years	Not mentioned	Post-op

5	Kim et al. [1]	78	Male	Palpable mass in the right upper thigh and swelling of the right lower extremity for 2 months	40 years ago	Open	Post-op

6	Sameera and Sohil [11]	51	Male	Complaints of flank pain and hematuria	Not mentioned	Not mentioned	Pre-op

7	Korkolis et al. [3]	49	Female	6-month history of vague right lower quadrant pain radiating through to the back	25 years previously	Not mentioned	Post-op

8	Lien et al. [2]	66	Male	Gradual onset of right lower quadrant (RLQ) pain	30 years	Not mentioned but presumably open	Post-op

9	Lien et al. [2]	45 years	Female	3-day history of RLQ pain associated with low-grade fever (37.8°C) and diarrhea	10 years previously	Not mentioned (but they mentioned surgical scar, it looks open)	No pre-op diagnosis

10	Yeong et al. [12]	54 years	Male	Painless abdominal mass which he first noticed 5 years ago, gradually increasing in size	Appendicectomy for an appendiceal abscess 25 years previously. The perforated appendix was in the prececal position, adherent to the terminal ileum and covered by omentum	Not mentioned but it looks open	Pre-op diagnosis of mesenteric or reduplication cyst was suggested

**Table 2:** Investigations, surgical procedure, histopathology, and outcomes.

Author	Investigations	Surgical procedure	Histopathology	Outcome
Rasch and Strange [8]	Barium enema: smooth deformity of the inferomedial border of the cecum with pressure on the terminal ileum	Exploratory laparotomy and right hemicolectomy	Cecal wall thickened with connective and muscular tissue Large amounts of mucous material, occurring in lakes of considerable size Fibrosis and occasional groups of normal mucus secreting cells	Discharged after 2 weeks

Ozgür et al. [6]	CT abdomen (hypoattenuating, 10∗9∗8 cm mass lying in the retrocecal region) US abdomen 10 × 9 × 8 cm mass with multiple echogenic layers in a wavy pattern filling the entire lesion, with thin but smooth wall	Exploratory laparotomy (9 × 7 × 3 cm mass and a fixed tumor adherent to the abdominal wall, posterior to the cecum Right hemicolectomy	Firm mass of 9 × 7 × 1.5 cm near the ileocecal region Normal colonic mucosa papillary projections of the mucinous adenocarcinoma lining the appendiceal mucosa	No follow-up

Cama Jitoko K [21]	US scan: normal	Diagnostic laparoscopy cystic lesion arising from the base of the stump of appendix 3 × 2 cm, excised laparoscopically	Periappendicitis with chronic inflammation and fibrosis	No follow-up

El Ajmi M, 2009 [10]	US scan: hypoechogenic mass 90 × 65 × 55 mm CT: retroperitoneal cystic structure measuring 13 × 58 cm, without mural calcification	Laparotomy Retroperitoneal cystic mass right ileocecal resection with sufficient margins	Mucinous cystadenoma of the appendiceal stump Five lymph nodes examined were free from the tumor Margins of resection and cytology were negative	Discharge on the 4^th^ postoperative day

Kim et al. [1]	Doppler US : a well-defined lobulated mass (10∗6∗30 cm) in the right lower abdomen extending into the right upper thigh CT : elongated, cystic mass in the extraperitoneal space of the right lower quadrant extending into the right thigh along the right femoral canal, indenting medial aspect and possibly originating from the cecum (i.e., the expected location of the appendix)	Two separate incisions, the cecum, terminal ileum, and a portion of the mass above the right femoral canal were removed with an ileocolostomy Mass below the right femoral canal was removed separately	Mucinous cystadenoma	No evidence of recurrence 3 years after surgery

Sameera and Sohil [11]	Ultrasound: 6 cm cystic lesion around the inferior pole of the right kidney CT No lesion in kidney but a tubular cystic structure in the right flank with inferior tip at the same site of previously inflamed appendix stump base, mucocele of stump	Exploratory laparotomy and mucocele resected	Benign mucinous cystadenoma	The patient was discharged well

Korkolis et al. [3]	CT : elliptical, 7 × 5 cm cystic mass, lying at the inferior aspect of the cecum; the lesion had smooth walls, scattered mural calcifications, and no surrounding inflammation	Exploratory laparotomy, well-encapsulated and calcified tumor, 8 × 5.5 × 4 cm in size, at the base of originating from the one cm, unburied, appendiceal stump Limited cecal resection with the mass	Histopathology revealed crowded, villotubular structures, with mild to moderate epithelial atypia together with acellular mucin pooling (75 ml mucin) No evidence of malignancy Appendiceal stump mucocele associated with a benign mucinous cystadenoma	Discharged home on the fifth postoperative day 18 months after surgery, free of symptoms No recurrence

Lien et al. [2]	US : dumbbell-shaped, heterogeneous cystic mass with internal echoes, 5 × 5 cm in diameter in the right lower abdomen CT demonstrated a cystic mass at the inferior aspect of the cecum	Right hemicolectomy; an 8.3^∗^ 6^∗^3.5 cm dumbbell-shaped tumor containing yellowish mucus originating from the 1 cm unburied appendiceal stump	Mucinous cystadenoma	Uneventful postoperative course No recurrence at 2 years

Lien et al. [2]	US showed an elongated cystic mass with internal echoes CT : well-defined cystic lesion without surrounding inflammation adjacent to the cecum and displacing the terminal ileum	The patient refused surgical intervention	None	She has remained well during the subsequent 4 years

Yeong, [16]	CT : intraperitoneal cystic mass in the right upper quadrant with peripheral calcification	Laparotomy Mass adherent to terminal ileum, cecum, and ascending colon The right iliac fossa contained scattered grey, mucoid nodules up to 0.4 cm in diameter Cyst not separated from the colon Right hemicolectomy	Mucocele due to a papillary cystadenocarcinoma arising in an appendiceal stump and associated with peritoneal metastases	Not mentioned

## Data Availability

The data used to support the findings of this study are included within the article.
